# Higher dietary polyphenol intake is associated with reduced pain sensitivity and migraine-related disability: A cross-sectional analysis

**DOI:** 10.1371/journal.pone.0351122

**Published:** 2026-06-08

**Authors:** Gabriele Bertotti, Alberto Roldán-Ruiz, Jaime Rodríguez-Vico, Andrea Gómez-García, Paula Marrero-Fernández, Miguel López-Moreno

**Affiliations:** 1 Dieta, Salud Planetaria y Rendimiento, Facultad de Ciencias de la Salud, Universidad Francisco de Vitoria, Madrid, Spain; 2 Institute of Health and Sport Sciences, Faculty of Health Science, Universidad Francisco de Vitoria, Madrid, Spain; 3 Headache Unit, Neurology Department, Hospital Universitario Fundación Jiménez Díaz, Madrid, Spain; 4 Department of Chemical Engineering and Pharmaceutical Technology, University of La Laguna, Santa Cruz de Tenerife, Canary Islands, Spain; Japanese Academy of Health and Practice, JAPAN

## Abstract

**Introduction:**

Migraine is a highly disabling neurological disorder characterized by recurrent headache attacks and altered pain processing. Emerging evidence suggests that dietary patterns rich in antioxidant compounds may modulate migraine-related pathophysiology, yet the specific association between polyphenol intake and objective pain sensitivity measures remains unclear. This study aimed to examine the association of dietary polyphenol with pain sensitivity and migraine-related disability in individuals with migraine.

**Materials and methods:**

This cross-sectional study including 72 adults (65 women) with a confirmed migraine diagnosis according to ICHD-III criteria. Participants completed a 3-day dietary record, and polyphenol intake was estimated using the Phenol-Explorer database. Clinical outcomes included pressure pain thresholds (PPTs) at trigeminal and extratrigeminal sites, migraine-related disability (MIDAS), perceived stress (PSS), and handgrip strength. Associations were analyzed using Pearson correlations and multiple linear regression models adjusted for age, sex, BMI, energy intake, and comorbidities.

**Results:**

Participants with longer disease duration reported higher polyphenol intake (p < 0.05). Total polyphenol intake was positively correlated with PPTs at the masseter (r = 0.27) and middle temporalis (r = 0.31), and inversely correlated with MIDAS scores (r = −0.25, all p < 0.05). In adjusted regression analyses, higher polyphenol intake predicted increased PPTs (masseter: β = 0.33; middle temporalis: β = 0.38) and lower MIDAS scores (β = −0.25, all p < 0.01). No associations were observed with handgrip strength or perceived stress.

**Conclusions:**

Higher dietary polyphenol intake was associated with reduced pain sensitivity and lower migraine-related disability, suggesting a potential role for polyphenol-rich diets in migraine management. Further randomized controlled trials are needed to confirm these associations and explore the mechanisms through which polyphenol-rich diets may influence migraine-related outcomes.

## Introduction

Migraine is a primary headache typically characterized by recurrent attacks of severe headache that are often highly disabling. These episodes can last between 4 and 72 hours, and they are commonly accompanied by nausea, vomiting, photophobia and phonophobia, and a range of additional physical, cognitive, and psychological symptoms [1]. Beyond these symptomatic manifestations, growing evidence indicates that clinical characteristics related to pain processing, such as pressure pain thresholds (PPTs) and conditioned pain modulation, are altered in patients with migraine [2,3]. These individuals often exhibit heightened pain intensity, although this may normalize between migraine episodes [4]. Migraines affect approximately 15% of the global population [5], and it represents not only a major health problem but also a substantial economic burden for society. In 2010, the annual costs of brain disorders in the European Union were estimated, showing that migraine alone accounts for more than 18 billion euros in direct and indirect expenses, primarily due to work loss [6]. Indeed, it has been estimated that patients with migraine generate 1,7 higher costs for the Spanish Health System than non-migraine matched controls [7].

Similarly, there is growing evidence suggesting that migraine may reflect a disturbance in brain energy metabolism, representing either a response to cerebral energy deficiency or a homeostatic compensation for excessive oxidative stress [8]. Understanding migraine through this metabolic framework may open new therapeutic opportunities targeting these underlying mechanisms. Accordingly, individuals with migraine have been found to exhibit distinct dietary patterns compared to healthy populations. Higher intakes of total and saturated fat observed in individuals with migraine may promote systemic inflammation, impair cerebral energy metabolism, and contribute to mitochondrial dysfunction, ultimately influencing migraine frequency and severity [9].

In line with this, although diet shows potential as a therapeutic approach for modulating the metabolic and inflammatory disturbances associated with migraine, current evidence remains limited and is largely focused on interventions such as dietary approaches to stop hypertension (DASH) and ketogenic diets [10]. Given the role of oxidative stress in migraine pathophysiology, dietary patterns rich in antioxidants compounds may also be relevant, as they could help counteract these metabolic and inflammatory disturbances and offer an additional non-phamacological strategy for migraine prevention and treatment. In this regard, diets rich in phytochemicals and polyphenols have been associated with lower severity of migraine-related symptoms [11]. Plant-based foods provide a wide range of bioactive compounds, including polyphenols, which exhibit antioxidant properties by modulating reactive oxygen species levels [12].

Taken together, these findings suggest that dietary patterns, particularly the rich in polyphenol-containing foods, may influence migraine-related pathophysiological processes. However, although overall diet quality has been linked to migraine burden, the specific association between polyphenol intake and migraine severity, including both clinical and psychophysical outcomes, remains unexplored. Investigating the relationship between polyphenol consumption and measures of pain perception may therefore provide new insights into dietary determinants of migraine and inform targeted nutritional strategies for its prevention and management.

The primary objective of this study was to examine the association between dietary polyphenol intake with lower pain sensitivity and reduced migraine-related disability in individuals with migraine. As a secondary objective, we aimed to explore whether polyphenol intake was also related to handgrip strength, and perceived migraine-related disability and stress.

## Methods

### Study design and participants

This cross-sectional observational study was conducted between 01/05/2024 and 22/11/2024. The sample size was previously calculated through the G-Power program. Given the absence of prior studies evaluating the association between PPTs and polyphenol intake in this population, a sensitivity analysis was performed testing small (r = 0.1), moderate (r = 0.3), and large (r ≥ 0.5) correlations [13]. A moderate correlation (r = 0.3) was selected as the most plausible and methodologically conservative estimate, in line with correlations reported in studies examining the relationship between polyphenol intake and migraine severity. Therefore, assuming a moderate Pearson correlation (r = 0.30), a one-sided α = 0.05 was used, reflecting the a priori hypothesis that higher polyphenol intake would be associated with lower pain sensitivity. With 80% statistical power, the required sample was estimated at 64 participants. To account for a potential 20% dropout rate, 71 participants were ultimately enrolled.

Participants were recruited from the Headache Specialized Unit of the Neurology Department of Hospital Universitario Fundación Jiménez Díaz (Madrid, Spain). Participants were eligible if they were 20–65 years old and had a confirmed diagnosis of migraine, including migraine with or without aura and chronic migraine, according to the International Classification of Headache Disorders, 3rd Edition (ICHD-III) [1] as assessed by an experienced neurologist. Individuals undergoing preventive pharmacological migraine treatment were allowed to participate. Participants were excluded if they were experiencing an acute migraine attack at the time of assessment, had probable migraine, any other primary or secondary headache disorder, or had taken abortive or muscle relaxant medication within 48 hours prior to testing. Additional exclusion criteria included dementia or severe psychiatric disorder, conditions affecting comprehension or participation, fibromyalgia, a history of cranial, cervical, or lumbar surgery, rheumatologic, systemic, or other neurological diseases, or adherence to a dietary regimen specifically aimed at modifying nutritional intake or body composition.

All participants were informed of the study objectives, and written informed consent was obtained prior to inclusion. The followed the guidelines established in the Declaration of Helsinki, and all procedures were reviewed and approved by the Ethics Committee of the Hospital Universitario Fundación Jiménez Díaz (10/23).

### Study procedures

The study comprised a single visit conducted at the Headache Specialized Unit of the Neurology Department of Hospital Universitario Fundación Jiménez Díaz (Madrid, Spain). During this session, participants completed a brief questionnaire collecting basic demographic information (age, sex) and migraine-related characteristics. These include migraine attacks frequency, workdays lost in the last 3 months, and headache pain intensity. Headache frequency was estimated with headache diaries and expressed as migraine days per month, while headache pain intensity was measured with the Numerical Pain Rating scale (NPRS), which consists of an 11-point scale ranging from 0 (no pain) to 10 (worst pain imaginable). It has demonstrated good test–retest reliability (Intraclass Correlation Coefficient = 0.63–0.92) [14–16].They then received detailed instructions on completing a 3-day dietary record, which included two weekdays and one weekend day randomly assigned by the investigator. Participants returned the completed records afterward, ensuring prospective data collection.

### Questionnaires

1. **Migraine Disability**

Migraine-related disability was measured using MIDAS, a standardized tool developed to quantify the impact of migraine on daily functioning. The MIDAS questionnaire evaluates the number of days over the past three months in which migraine limited or prevented performance in work, household, and social activities. The total score is calculated by summing responses to five key items, classifying disability into four grades from minimal to severe. It demonstrates strong reliability and validity, making it one of the most widely used instruments for assessing migraine-related disability in both clinical and research contexts [17]. In this study, the Spanish validated version was used [18].

2. **Perceived Stress**

Perceived stress levels were evaluated using the (PSS), a widely used validated instrument designed to measure the degree to which individuals perceive their life situations as stressful. The PSS assesses how unpredictable, uncontrollable, and overloaded respondents find their lives during the past month. It consists of 10 items rated on a 5-point Likert scale ranging from 0 (“never”) to 4 (“very often”), with higher scores indicating greater perceived stress. This tool has been validated in the Spanish population, and it has been shown to possess adequate reliability (internal consistency, alpha = .81, and test-retest, r = .73), validity (concurrent), and sensitivity [19].

### Anthropometric and functional measurements

Anthropometric measurements were performed using calibrated Tanita BC-601 (Tanita Corporation, Japan). Body weight was measured in kilograms with participants barefoot and lightly clothed, rounded to the nearest 0.1 kg. Height was measured with participants standing fully erect, feet together, head in the Frankfort plane, and arms hanging freely at the sides, rounded to the nearest 0.1 cm. Body mass index (BMI) was calculated as weight (kg) divided by height squared (m²). Participants were classified according to BMI as underweight (<18.5 kg/m²), normal weight (18.5–24.9 kg/m²), overweight (25–29.9 kg/m²), or obese (≥30 kg/m²), following World Health Organization (WHO) criteria [20].

Handgrip strength was measured using a Jamar hand dynamometer (Sammons Preston Rolyan, Bolingbrook, IL). This device has demonstrated good to excellent intra-rater reliability for the assessment of isometric handgrip strength (Intraclass Correlation Coefficient = 0.85–0.98) [21]. The mean of three consecutive measurements with 30-second intervals between trials were expressed in kilogram-force (kgf), on both hands. This assessment has shown excellent reliability across different populations and clinical conditions [22,23].

### Clinical variables

The primary measures included in this study were clinical variables commonly used to assess pain sensitivity and related outcomes in patients with migraine [24,25]. PPTs were assessed with a Digital Force Gauge SF-500 algometer. PPTs are defined as the minimal level of pressure that provokes the first sensation of pain [26]. The algometer consists of a pressure meter equipped with a 1 cm² rubber tip. This instrument has demonstrated high reliability for quantifying PPTs (ICC = 0.91, 95% CI: 0.82–0.97) [27]. Participants were seated comfortably while progressively increasing pressure was applied at a rate of 50 kPa/s [28]. They were instructed to immediately indicate when the pressure sensation first became painful. PPTs were recorded bilaterally at three trigeminal sites, corresponding to the masseter muscle and the anterior and middle fibers of the temporalis muscle. Additionally, extratrigeminal sites included the sternocleidomastoid, upper trapezius, C2–C3 zygapophyseal joints, and the thenar eminence [29]. The order of assessment was randomized across participants [30]. At each anatomical location, three consecutive measurements were obtained with 30-second intervals between repetitions [28]. The mean value of the three trials was calculated for each site and side. Data were expressed in kg/cm² by calculating the mean results of both sides.

### Polyphenol intake estimation

Polyphenol intake was estimated from the previously completed three-day dietary records, which included two non-consecutive weekdays and one weekend day. Each food item consumed was extracted and averaged over the three days, and then matched to corresponding foods in the Phenol‑Explorer web-based database (www.phenol-explorer.eu), which provides mean content values for over 500 individual polyphenols across more than 400 food items [31]. Matching followed a hierarchical approach: when participants reported a specific variety or cultivar, the corresponding variety-specific entry was used; otherwise, the generic food entry was selected. For foods reported as cooked or processed, the corresponding cooked or processed entry was preferentially selected when available in the database; when only the raw food entry was available, this was used without further correction by retention factors. For each food item, the consumption amount (g or mL) was multiplied by the polyphenol content (mg per g/mL) from the database. Individual polyphenol intakes were then summed to obtain estimates of total polyphenol intake as well as intakes of specific polyphenol classes, including flavonoids, phenolic acids, lignans and stilbenes. This approach allowed for a quantitative and reproducible estimation of individual and total polyphenol intake [32].

### Statistical analysis

Quantitative variables are presented as mean ± standard deviation (SD), and categorical variables as number (percentage). Differences in quantitative variables across tertiles of total phenolics intake were assessed using one-way analysis of variance (ANOVA), while the distribution of categorical variables across tertiles was evaluated with the Chi-square test. Pearson’s correlation coefficients were first calculated to examine the relationships between total polyphenol intake and PPTs, MIDAS and PSS-14 scores, and handgrip strength. Correlation analyses were considered exploratory; therefore, no formal correction for multiple comparisons was applied. Variables showing significant correlations were then included in multiple linear regression analysis, with beta (β) estimates and 95% confidence intervals (CI) being reported. In Model 1, analyses were adjusted for age, sex, and total energy intake. Model 2 further adjusted for body mass index, dietary energy intake, other comorbidities, preventive medication use within the 48 hours before assessment, and time since the last acute migraine attack. Statistical analyses were conducted using R statistical software (version 4.5.2) and RStudio (version 2025.09.2 + 418). A p-value < 0.05 was considered statistically significant.

## Results

**Fig 1:**
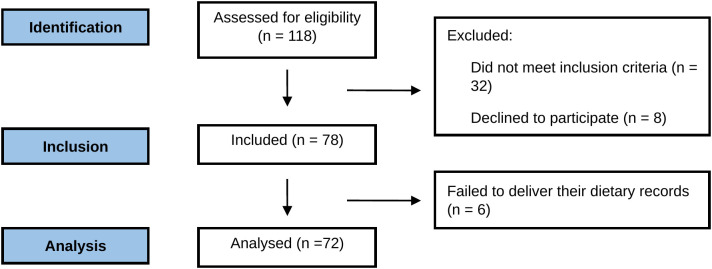
Flow chart of participants.

A total of 118 patients were screened for eligibility, of whom 78 met the inclusion criteria and were included. The final analysis included 72 patients (65 females and 7 males) due to 6 participants failing to deliver their dietary records (Fig 1). Participants had a mean age of 46.58 (10.79) years and a mean BMI of 25.22 (5.03) kg/m². On average, they reported 33.40 (43.56) migraine attacks and 4.49 (15.51) workdays lost due to migraine in the last 3 months, with a mean time since diagnosis of 16.87 (14.23) years. Pain intensity was 7.14 (1.30), and participants had experienced 8.05 ± 11.39 days since their last migraine attack.

When participants were grouped into tertiles according to total phenolic intake (Table 1), no significant differences were observed among subgroups for demographic, clinical, or body composition variables. However, years since migraine diagnosis differed significantly across tertiles, with higher polyphenol intake reported among participants with longer disease duration (*p* < 0.05).

**Table 1:** Characteristics of the study population stratified by tertiles (T) of total phenolics intake.

	Terciles of total phenolics intake			F	P-value
	T1 (N = 24)	T2 (N = 24)	T3 (N = 24)		
Age	44.5 (9.9)	46.6 (11.6)	49.4 (10.0)	1.291	0.281
Females	21 (87.5)	21 (87.5)	23 (95.8)	0.617	0.542
Migraines attacks in the past 3 months	31.6 (29.4)	40.8 (66.3)	28.7 (24.1)	0.495	0.611
Workdays lost in the past 3 months	6.2 (18.5)	2.3 (8.3)	5.1 (18.2)	0.376	0.688
Years since diagnosis	14.8 (14.4)	13.1 (12.4)	23.2 (14.2)	3.756	**0.028**
Energy intake (kcal/day)	2434 (130)	2398 (156)	2221 (132)	0.298	0.783
Body weigth	70.2 (14.5)	70.7 (18.9)	68.7 (15.1)	0.971	0.384
Fat free mass (kg)	42.4 (6.1)	45.3 (8.3)	41.5 (5.1)	2.211	0.117
Fat mass (%)	34.4 (9.3)	31.7 (10.8)	31.9 (1.7)	0.573	0.566
Visceral fat	7.3 (4.5)	7.2 (4.3)	6.5 (4.1)	0.245	0.784
Bone mass (kg)	2.3 (0.3)	2.3 (0.4)	2.4 (0.8)	0.312	0.733
BMI	25.9 (5.4)	25.6 (5.8)	24.1 (3.7)	0.909	0.408

Data are presented as mean (Standard deviation) or number (% within tertiles of total phenolics intakes). p-values were obtained using chi-square tests for categorical variables and one-way analysis of variance (ANOVA) for continuous variables. A p-value < 0.05 was considered statistically significant. BMI, body mass index.

### Correlation analyses of polyphenol intake with pain and functional measures

Pearson’s correlation coefficients were used to examine the relationships of total polyphenol intake with PPTs, MIDAS and PSS-14 scores, and handgrip strength (Tables 2 and 3). Significant associations were observed between PPTs and total polyphenols intake at the masseter (r = 0.27; p < 0.05), middle temporalis (r = 0.31; p < 0.05), and upper trapezius muscles (r = 0.27; p < 0.05). Total polyphenol intake was also significantly correlated with MIDAS score (r = −0.25; p < 0.05), with higher intake corresponding to lower migraine-related disability (Table 3).

**Table 2:** Pearson’s correlation coefficients of pain pressure thresholds and polyphenol intake.

	Flavonoids	Phenolics Acids	Lignans	Stilbens	Other Polyphenols	Total Polyphenols
Masseter	0.190	0.204	0.016	0.042	0.110	0.268*
Anterior Temporalis	0.145	0.260	−0.002	−0.190	−0.004	0.080
Middle Temporalis	0.229	0.042	−0.014	−0.176	0.098	0.305*
Sternocleidomastoid	0.164	0.077	−0.023	−0.156	0.125	0.167
Upper trapezius	0.187	0.12	0.24	0.171	0.089	0.271*
C2-C3 joint	0.127	−0.04	−0.022	0.105	−0.007	0.021
Thenar eminence	0.136	0.146	−0.061	−0.07	−0.033	0.139

Pearson correlation. * p value < 0.05.

**Table 3:** Pearson’s correlation coefficients of MIDAS/PSS14, handgrip strength and phenolic contents.

	Flavonoids	Phenolics Acids	Lignans	Stilbens	Other Polyphenols	Total Polyphenols
MIDAS	0.029	−0.224	−0.079	0.038	−0.213	−0.248*
PSS14	−0.001	0.044	0.136	−0.059	0.063	0.027
Dominant handgrip	−0.052	0.182	0.114	0.030	0.035	0.137
Non-dominant handgrip	−0.019	0.171	0.086	0.092	0.138	0.184

Pearson correlation. * p value < 0.05.

### Regression analyses of polyphenol intake and clinical outcomes

**Fig 2:**
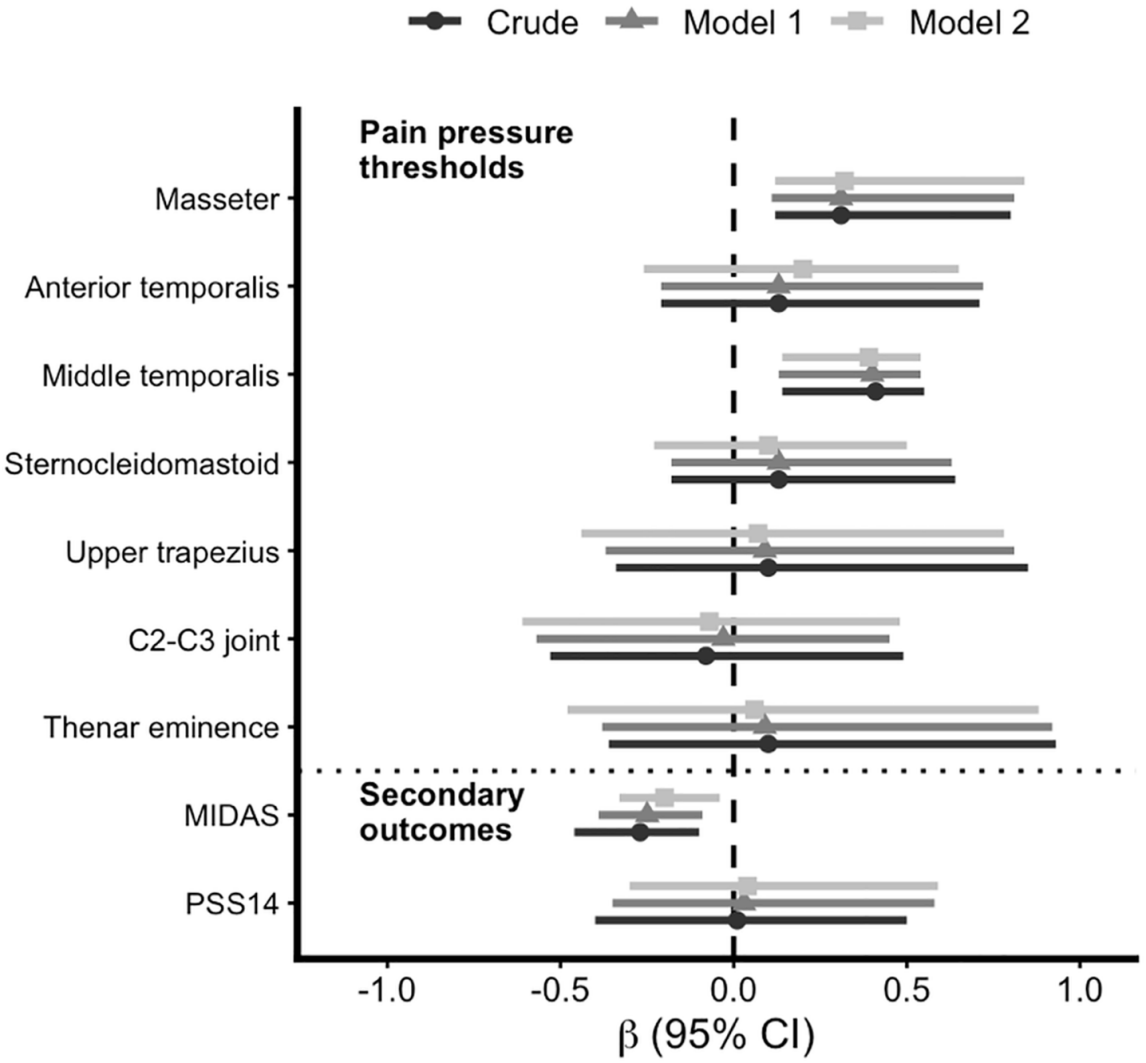
Forest plot of crude and adjusted associations between total polyphenol intake tertiles and migraine-related clinical outcomes.

Based on the significant correlations observed, multiple linear regression analyses were performed to examine the predictive value of total polyphenols intake on PPTs, and migraine-related disability. Fig 2 provides a graphical representation of crude and adjusted regression coefficients for all evaluated outcomes. After adjusting for potential confounders (age, sex, BMI, energy intake, and comorbidities), higher polyphenol intake remained a significant predictor of increased PPTs in the masseter (β = 0.32, 95% CI 0.12–0.84, p = 0.008) and middle temporalis muscles (β = 0.39, 95% CI 0.14–0.54, p = 0.002) (Table 4). Multiple linear regression analyses examining the predictive value of total polyphenol intake on migraine-related outcomes showed that higher polyphenol intake significantly predicted lower MIDAS scores (β = −0.20, 95% CI −0.33 to −0.04, p = 0.009) after adjustment for age, sex, BMI, energy intake, and comorbidities (Table 5).

**Table 4:** Beta (*β*) and 95% confidence interval for pain pressure thresholds outcomes according to tertiles (T) of total polyphenols.

	Tertiles of total polyphenols			
	T1	T2	T3	p-value
**Masseter**				
Crude	Ref	−0.07 (−0.46, 0.45)	**0.31 (0.12, 0.80)**	0.007
Model 1	Ref	−0.07 (−0.46, 0.25)	**0.31 (0.11, 0.81)**	0.009
Model 2	Ref	−0.06 (−0.44, 0.23)	**0.32 (0.12, 0.84)**	0.008
**Anterior temporalis**				
Crude	Ref	−0.04 (−0.54, 0.38)	0.13 (−0.21, 0.71)	0.295
Model 1	Ref	−0.03 (−0.52, 0.40)	0.13 (−0.21, 0.72)	0.275
Model 2	Ref	−0.01 (−0.47, 0.42)	0.80 (−0.26, 0.65)	0.498
**Middle temporalis**				
Crude	Ref	0.001 (−0.49, 0.49)	**0.41 (0.14, 0.55)**	0.001
Model 1	Ref	0.01 (−0.46, 0.50)	**0.40 (0.13, 0.54)**	0.001
Model 2	Ref	0.00 (−0.48, 0.49)	**0.39 (0.14, 0.54)**	0.002
**Sternocleidomastoid**				
Crude	Ref	−0.09 (−0.57, 0.25)	0.13 (−0.18, 0.64)	0.397
Model 1	Ref	−0.08 (−0.54, 0.27)	0.13 (−0.18, 0.63)	0.572
Model 2	Ref	−0.04 (−0.50, 0.31)	0.10 (−0.23, 0.50)	0.581
**Upper trapezius**				
Crude	Ref	−0.02 (−0.59, 0.59)	0.10 (−0.34, 0.85)	0.329
Model 1	Ref	0.01 (−0.55, 0.62)	0.09 (−0.37, 0.81)	0.364
Model 2	Ref	0.00 (−0.58, 0.60)	0.07 (−0.44, 0.78)	0.499
**C2-C3 joint**				
Crude	Ref	−0.07 (−0.65, 0.37)	−0.08 (−0.53, 0.49)	0.678
Model 1	Ref	−0.05 (−0.61, 0.39)	−0.03 (−0.57, 0.45)	0.594
Model 2	Ref	−0.03 (−0.57, 0.42)	−0.07 (−0.61, 0.48)	0.488
**Thenar eminence**				
Crude	Ref	−0.01 (−0.68, 0.61)	0.10 (−0.36, 0.93)	0.346
Model 1	Ref	0.00 (−0.64, 0.64)	0.09 (−0.38, 0.92)	0.345
Model 2	Ref	0.01 (−0.61, 0.64)	0.06 (−0.48, 0.88)	0.464

Data are presented as *β* (95% confidence interval) and obtained from linear regression. Model 1: Adjusted for age and sex. Model 2: Adjusted age, sex, body mass index, energy dietary intake, other comorbidities, abortive medication use within the 48 hours before assessment, and time since the last acute migraine attack.

**Table 5:** Beta (*β*) and 95% confidence interval for MIDAS/PSS14 of migraine headaches according to tertiles (T) of total polyphenols.

	Tertiles of total polyphenols			
	T1	T2	T3	p-value
**MIDAS**				
Crude	Ref	0.10 (−0.23, 0.45)	**−0.27 (−0.46, −0.10)**	0.001
Model 1	Ref	0.09 (−0.25, 0.40)	**−0.25 (−0.39, −0.09)**	0.005
Model 2	Ref	0.10 (−0.25, 0.40)	**−0.20 (−0.33, −0.04)**	0.009
**PSS14**				
Crude	Ref	0.05 (−0.30, 0.50)	0.01 (−0.40, 0.50)	0.789
Model 1	Ref	0.05 (−0.29, 0.53)	0.03 (−0.35, 0.58)	0.850
Model 2	Ref	0.05 (−0.29, 0.56)	0.04 (−0.30, 0.59)	0.802

Data are presented as *β* (95% confidence interval) and obtained from linear regression. Model 1: Adjusted for age and sex. Model 2: Adjusted age, sex, body mass index, energy dietary intake, other comorbidities, abortive medication use within the 48 hours before assessment, and time since the last acute migraine attack.

## Discussion

The primary objective of this study was to examine whether dietary polyphenol intake was associated with pain sensitivity and migraine-related disability in individuals with migraine. In this cross-sectional analysis, we found that higher total polyphenol intake was consistently associated with lower pain sensitivity, as evidenced by higher PPTs at the masseter, middle temporalis, and upper trapezius muscles. Additionally, an inverse association was observed with migraine-related disability, with higher polyphenol intake corresponding to lower MIDAS scores. These associations remained significant after adjustment for demographic, dietary, and clinical factors. By contrast, no associations were identified between polyphenol intake and handgrip strength or PSS-14, and no specific polyphenol subclasses were related to the outcomes assessed.

Our findings showed that higher total polyphenol intake was associated with lower pain sensitivity and migraine-related disability. These findings align with a growing body of evidence indicating that dietary patterns rich in antioxidant and polyphenolic compounds may contribute to lower migraine burden. In line with this, diets with higher phytochemical and polyphenol content, particularly flavanones and lignans, have been associated with lower migraine severity [33], Importantly, that study was based on subjective assessments of migraine severity (e.g., MIDAS, VAS), while our study extends this evidence by incorporating objective indicators, such as PPTs, together with clinical variables. Similarly, higher dietary antioxidant quality scores have been associated with lower migraine intensity and frequency in women and specific antioxidants such as vitamin C were inversely associated with pain severity [34]. Supporting this, a recent study found a linear inverse association between dietary vitamin C intake and migraine prevalence, further reinforcing the potential role of antioxidant nutrients in headache prevention [35]. Likewise, another article demonstrated that a higher composite dietary antioxidant index, reflecting cumulative intake of vitamins A, C, E, selenium, zinc, and carotenoids, was associated with a reduced risk of severe headache or migraine [36]. This collective evidence reinforces the potential relevance of antioxidant- and polyphenol-rich dietary patterns in migraine management. This may be of particular interest in light of recent data from Spain showing that individuals with migraine consume fewer plant-based foods than recommended by national dietary guidelines (AESAN), while exceeding recommended meat intake [37].

Several biological mechanisms may support the observed associations between higher polyphenol intake, lower pain sensitivity, and reduced migraine-related disability. Through their antioxidant and anti-inflammatory properties [38], higher polyphenol intake may help mitigate oxidative stress, which has been shown to exert pronociceptive effects in the meninges, partly through activation of transient receptor potential ankyrin 1 (TRPA1) [39], processes that might be linked to migraine pathophysiology. Moreover, evidence from experimental and translational studies indicates that polyphenolic compounds modulate neuroinflammatory signalling cascades. Specifically, they downregulate key pathways, including nuclear factor-κB (NF-κB), phosphoinositide-3-kinase/protein kinase B (PI3K/AKT), Janus kinase/signal transducer and activator of transcription (JAK/STAT), and mitogen-activated protein kinase (MAPK), thereby reducing the production of proinflammatory cytokines [40]. In addition, these compounds may suppress activation of microglia, astrocytes, mast cells, and other neuroimmune cells implicated in sensitization processes relevant to migraine [41]. From a neurobiological perspective, it is plausible to hypothesize that dietary patterns rich in pro-inflammatory components, or low in antioxidant and polyphenolic compounds, could exacerbate central sensitization through oxidative stress and neuroinflammatory pathways. Nevertheless, this hypothesis requires confirmation through rigorously designed randomized controlled trials.

In addition to pain-related outcomes, other clinical characteristics such as physical function may contribute to the overall migraine profile. Reduced handgrip strength has been recently observed in patients with migraine [42], suggesting that functional impairments may accompany the sensory and disability-related alterations commonly described in this population. In our sample, handgrip strength was not associated with total or specific polyphenol intake, indicating that the influence of dietary phenolic compounds may be more directly linked to nociceptive processing rather than to general physical performance measures. Similarly, perceived stress levels assessed through the PSS were not significantly associated with polyphenol intake, suggesting that psychological factors may not mediate this dietary relationship. In contrast, the only medical record-related symptom that was significantly associated with higher phenolic compound intake was years since migraine diagnosis, being this found among participants with a longer disease duration. This finding may indicate that individuals with a longer history of migraine become increasingly aware of the potential role of healthy habits in managing their condition, which could lead to a gradual adoption of healthier eating patterns, including a higher intake of phenolic compounds.

### Strengths and limitations

The present study has several strengths, including the use of clinically relevant outcomes such as PPTs and the inclusion of migraine-related disability assessed through a widely used and validated instrument (MIDAS). Similarly, the use of multiple linear regression analyses adjusted for potential confounders strengthens the reliability of the observed associations. For instance, preventive migraine treatment use was included as a covariate in adjusted models to reduce potential confounding related to medication status. Additionally, polyphenol intake was estimated using the Phenol-Explorer database, allowing for a quantitative and reproducible assessment of total and specific polyphenol classes.

However, several limitations should be acknowledged. The cross-sectional design prevents causal inferences, and residual confounding cannot be fully excluded despite adjustment for key covariates. The sample size was relatively modest and predominantly female, which may also limit generalizability. Although dietary records provide detailed intake information, they rely on self-reports and may be subject to reporting bias. Moreover, estimated polyphenol intake does not account for individual variability in absorption, metabolism, or bioavailability. Polyphenol exposure derived from food composition databases is also subject to compositional variability arising from cultivar, storage, culinary, and industrial processing factors that are not fully captured by 3-day dietary records, and retention factors for cooking-related losses were not applied when only raw entries were available. Additionally, preventive migraine treatment use was included as a covariate in adjusted models to reduce potential confounding related to medication status. Other nutrients or bioactive compounds present in the diet, beyond polyphenols, may also influence pain sensitivity and migraine-related outcomes, which could contribute to residual confounding.

## Conclusions

Higher dietary polyphenol intake was associated with reduced pain sensitivity and lower migraine-related disability, suggesting a potential role for polyphenol-rich diets in modulating migraine outcomes. These findings highlight the potential utility of polyphenol-rich dietary strategies as complementary approaches for migraine management. Randomized controlled trials are warranted to establish causality and further explore the underlying biological mechanisms.

## Supporting information

S1 Data Data taking EN. (XLSX)
